# Succinate as a Regulator of Hepatic Stellate Cells in Liver Fibrosis

**DOI:** 10.3389/fendo.2018.00455

**Published:** 2018-08-21

**Authors:** Eun-Hee Cho

**Affiliations:** Department of Internal Medicine, School of Medicine, Kangwon National University, Chuncheon, South Korea

**Keywords:** succinate, GPR91, hepatic stellate cell, liver fibrosis, non-alcoholic fatty liver disease

## Abstract

The rapid increase of obesity rates worldwide is associated with chronic liver injury due to non-alcoholic fatty liver disease and non-alcoholic steatohepatitis. Chronic liver inflammation drives hepatic fibrosis, which is a highly conserved and coordinated protective response to tissue injury, and is a reversible process. Hepatocytes, immune cells, and hepatic stellate cells (HSCs) have been identified as key players in the mechanisms of hepatic fibrosis and inflammation. During the last decade, succinate, an intermediate of the tricarboxylic acid cycle in mitochondrial ATP production, has emerged as an important signaling molecule in various diseases. Succinate acts as an extracellular ligand for G-protein coupled receptor 91, also known as succinate receptor 1, which is mainly expressed in the kidney, heart, liver, immune cells, and retinal cells, suggesting a widespread function in cellular metabolism. Furthermore, succinate stabilizes hypoxia-inducible factor-1α in immune cells and tumors as a signaling molecule, and has been shown to post-translationally modify proteins. This review summarizes the recent evidence pointing to an additional role of succinate in profibrotic signaling, along with its downstream signaling pathways, and updates the current state of knowledge on the role of succinate in liver fibrosis through its action on HSCs. Further focus on this link can help identify succinate, its receptor, and its downstream signaling molecules as new targets for the treatment of liver fibrosis.

## Introduction

Liver fibrosis involves the excessive accumulation of extracellular matrix (ECM) proteins as the result of chronic liver damage due to chronic hepatitis B or C infection, alcohol abuse, non-alcoholic fatty liver disease (NAFLD), and free fatty acids accumulation (1). Although NAFLD, a manifestation of the hepatic complications of obesity, is a common liver disorder, its prevention and treatment remain significant challenges. Despite its high prevalence, only 5–10% of patients affected by NAFLD ultimately develop non-alcoholic steatohepatitis (NASH) with consequent progressive liver injury to advanced hepatic fibrosis, cirrhosis, portal hypertension, and liver cancer (2).

Liver injury causes necrosis and/or apoptosis of the parenchymal cells such as hepatocytes and cholangiocytes. The release of cell contents and reactive oxygen species (ROS) from damaged hepatocytes activates hepatic stellate cells (HSCs) and resident Kupffer cells (3). Activated Kupffer cells and HSCs phagocytose dead and apoptotic cells and also secrete pro-inflammatory cytokines which contribute to further hepatocyte injury, and chemokines such as the CC-chemokine receptor 2 (CCR2) which recruit a pro-fibrotic Ly-6C^hi^ monocytes population to the injured site (4, 5), where they develop into inflammatory and fibrogenic Ly-6C^hi^ macrophages. During chronic injury, these Ly-6C^hi^ macrophages activate HSCs to become collagen-producing myofibroblasts and promote the recruitment of monocytes, other inflammatory cells. If liver injury ceases, specific molecular signals trigger hepatic macrophages to switch their phenotype toward Ly-6C^lo^ restorative macrophages that promote tissue repair and regression of fibrosis (5).

More recently, attention is progressively shifting toward the pro-fibrotic microenvironment of the activated Ly-6C^hi^ macrophages during chronic injury as a switch cells to control the progression or resolution to induce the transition of resting HSCs into activated HSCs (6).

HSCs are considered to be the principal collagen-producing cells in the production of ECM proteins during persistent liver injury (7). In the normal liver, HSCs reside in the space of Disse in a quiescent form containing vitamin A. However, upon chronic liver damage, the HSCs activate or transdifferentiate into myofibroblast-like cells with contractile, migratory, proinflammatory, and fibrogenic properties, suggesting HSC activation as a central mechanism underlying liver fibrogenesis (8, 9).

Succinate is an intermediate of the tricarboxylic acid (TCA) cycle and plays a central role in ATP generation in the mitochondria. Since the identification of G-protein coupled receptor 91 (GPR91) as a succinate receptor, known as succinate receptor 1 (10), several reports have indicated that succinate is not only a central metabolite in the TCA cycle but also regulates various cell functions involved in epigenetics, tumorigenesis, signal transduction, inflammation, and paracrine modulation in various tissues, including the kidney, retina, heart, immune tissue, and liver (11–13). In particular, succinate has emerged as an important signaling molecule in the activation of HSCs (14), renin release in macula densa cells (15), exacerbation of rheumatoid arthritis (16), vascular endothelial growth factor release in the retina (17, 18), sensing immunological danger in dendritic cells (19), cardiac hypertrophy (20, 21), and osteoclastogenesis (22). This expanding evidence of functions for succinate suggests a broader role in cellular metabolism and human disease.

Accordingly, the aim of this review was to update the current state of knowledge on these multiple roles of succinate, with specific focus on its activity in HSCs toward identifying novel targets for the treatment of liver fibrosis.

## Succinate as a critical regulator of HSCs in liver fibrosis

As mentioned above, activation of HSCs is considered to be a critical event in the development of hepatic fibrosis (8, 23, 24). Once HSCs are activated in response to soluble stimuli such as PDGF, TGF-β, leptins, neuroendocrine signals (2-AG), TLR signaling, and VEGF (25), perpetuation of HSCs follows, characterized by proliferation, contractility, fibrogenesis, altered matrix degradation, chemotaxis, and inflammatory signaling (9). However, these processes contributing to liver fibrosis are known to be reversible, even at a late stage of the disease (26, 27), suggesting the possibility of an anti-fibrotic therapeutic strategy by regulating HSCs. Indeed, inhibition of HSCs activation initiation or suppression of the perpetuation of HSCs, such as proliferation and migration, chemotaxis, or inflammatory signaling, or enhancement of the apoptosis of HSCs may provide a novel drug targeted therapy for hepatic fibrosis (28, 29).

Succinate is normally present in the blood plasma at a concentration of ~2–20 μM (30, 31). He Weihai and colleagues demonstrated that the half-maximal response concentration for the succinate-induced activation of human GPR91 was 56 ± 8 μM, indicating that even slight elevation of the blood succinate concentration may completely activate GPR91 (10). However, a rapid increase in succinate levels up to the millimolar range (1,000–2,690 μM) has been demonstrated in pathological conditions, including in patients with coronary artery disease and following hepatic transplantation (20). This may suggest that GPR91 largely functions as a metabolic sensor of oxidative damage rather than as a physiological mediator of signaling (13).

With the discovery of the previously considered orphan GPR91 as a succinate receptor in 2004 (10), several studies have accumulated focusing on the role of GPR91 as a succinate receptor. GPR91 is highly expressed in the liver, kidney, and spleen (10). The group of Michael H. Nathanson first described the role of succinate and GPR91 in the liver (14). They suggested that the succinate receptor was only expressed in quiescent HSCs of rats, and ischemia of an isolated perfused liver induced by interrupting portal flow for 30 min led to an increased release of succinate by up to 14-fold, which activated HSCs (14). However, our study partly contradicted these previous findings by showing that GPR91 is overexpressed in activated HSCs, and GPR91 knockdown with small interfering RNA deactivated the HSCs, demonstrating that succinate caused HSCs activation through GPR91 signaling (32). Moreover, another study showed that GPR91 deletion protected mice from high-fat diet-induced obesity only during the initial period (33), suggesting that GPR91 might be an early sensor for dietary energy intake.

In hepatocytes, incubation of inner mitochondrial membrane (IMM) proteins with succinyl-CoA, another TCA intermediate, leads to global lysine succinylation, and sirtuin 5 (SIRT5) can reverse the succinyl-CoA-driven lysine succinylation of complex I by binding to cardiolipin on the IMM (34). However, SIRT5 was also found to suppress the pyruvate dehydrogenase complex and succinate dehydrogenase (35). Currently there is no concrete data on the role of succinate in mitochondria function and ROS production in hepatocytes suggesting the further research on this area.

**Figure 1:**
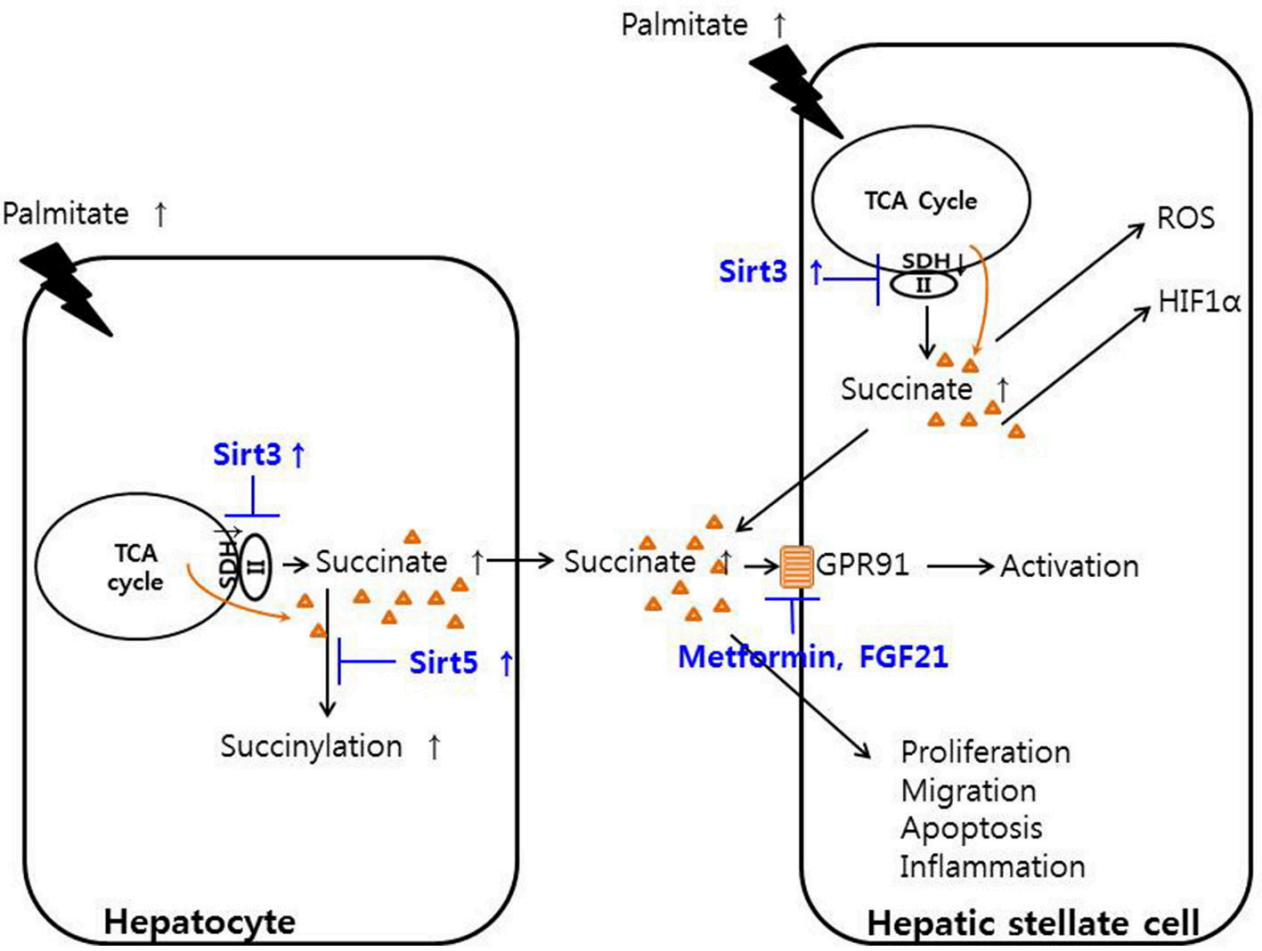
Function of succinate in hepatic stellate cells and hepatocytes. Palmitate decreased the activity of succinate dehydrogenase, thereby increasing succinate in hepatocytes and hepatic stellate cells. Increased cytoplasmic succinate goes out the cells and bind to GPR91 and activating hepatic stellate cells or regulate the proliferation, migration, apoptosis, and inflammation of hepatic stellate cells. SIRT3 activator or metformin or fibroblast growth factor 21 may regulates the hepatic stellate cells.

The overall functions and involvement of succinate in hepatocytes and HSCs are summarized schematically in Figure 1. Although HSCs were traditionally considered to mainly function as recipients of inflammatory signals, our recent studies show that HSCs also participate in pro-inflammatory action by producing some inflammatory cytokines, including interleukin-6 (IL-6) and tumor necrosis factor-alpha (TNF-α), but not TGF-β (36). Our team also demonstrated that succinate can induce the activation of HSCs, and regulate their proliferation, migration, and apoptosis *in vitro*, along with increasing the secretion of inflammatory cytokines such as IL-6 and TNF-α (36, 32).

SIRT3 is a major NAD^+^-dependent mitochondrial deacetylase and is highly expressed in the liver, brain, kidney, and skeletal muscle (37, 38). SIRT3 regulates succinate dehydrogenase (SDH) activity in the mitochondria by deactylation (39), and SIRT3 ablation in a *Sirt3*^−/−^ mouse line led to increased susceptibility for developing NASH (40).

SDH, also known as electron transport chain complex II, is part of both the TCA cycle and the respiratory electron transfer chain. Within the TCA cycle, SDH oxidizes succinate to fumarate (41). Increased SIRT3 activity up-regulates the activity of SDH, leading to a decrease in succinate concentrations; thus, inhibition of SDH activity results in the accumulation of succinate. One study showed that increasing SIRT3 expression in HSCs with adenoviral transfection or supplementation of resveratrol in methionine choline deficient (MCD) diet-fed mice led to a decrease in HSCs activation and alleviated hepatic fibrosis through the SDH-succinate-GPR91 pathway *in vivo* (42). The same study showed that hepatocytes pre-treated with palmitate contained and secreted high concentrations of succinate into the conditioned media, and their SIRT3 and SDH activities decreased compared with those of the control. In addition, the conditioned media from hepatocytes induced HSCs activation by increasing the succinate concentration along with GPR91 overexpression. In the MCD diet-fed animal model of liver fibrosis, metformin or fibroblast growth factor-21 treatment attenuated liver fibrosis by inhibiting succinate and GPR91 signaling, further suggesting the succinate-GPR91 complex as a therapeutic target of liver fibrosis (43, 44). Reducing the release of succinate from mitochondria, blocking of GPR91 in the membrane levels or SDH regulation by SIRT3 or de-succinylation might be a candidate targets but further study was needed to evaluate the exact down-steam pathway of succinate-GPR91 and discover the powerful exact drug target which may induce alleviation or regression of liver fibrosis.

With rapid advancements in the knowledge of GPR91 functions, several trials have been performed by various research groups to determine its clinical applications. The group of Dr. Bhuniya discovered the first selective GPR91 antagonist that may serve as a valuable test tool for establishing a proof-of-concept of the therapeutic efficacy of GPR91 antagonists in several animal disease models (45). However, no further advancement of this GPR91 antagonist has been reported to date. Recently, Trauelsen et al. (46) developed a novel synthetic GPR91 agonist that shows succinate-like action in human macrophages, providing a pharmacological tool to delineate the physiological and therapeutic actions of the GPR91-mediated functions of succinate through adopting a receptor structure-based approach.

## Conclusion and perspectives

Succinate is now emerging as an important signaling molecule in liver fibrosis. However, the translational research to improve therapeutics for the management of patients with liver fibrosis remains at an early stage. Although this research area is rapidly expanding, numerous limitations remain, including our incomplete understanding of the exact mechanism and role of succinate as a signaling molecule with regards to hepatocytes, immune cells, and HSCs in liver fibrosis *in vitro* and *in vivo*. The precise roles of the HIF-1α or GPR91 pathway, SDH, ROS metabolism, and succinylation with respect to the effects of succinate on liver fibrosis must be explored further. Furthermore, it is necessary to elucidate the critical point in the succinate pathway as a potential candidate for drug target selection. In conclusion, although some aspects of the functions of succinate and GPR91 are beginning to come to light, future challenges lie in delineating the cellular and molecular mechanisms responsible for these effects, and developing succinate-based therapies such as an effective GPR91 antagonist so as to prevent, cure, or ameliorate liver fibrosis.

## Author contributions

The author confirms being the sole contributor of this work and approved it for publication.

### Conflict of interest statement

The author declares that the research was conducted in the absence of any commercial or financial relationships that could be construed as a potential conflict of interest.

The reviewer JL and handling Editor declared their shared affiliation.
